# Personal

**Published:** 1909-10

**Authors:** 


### PERSONAL.

---

Dr. A. B. Miller, of Syracuse, was elected president of the American Association of Obstetricians an Gynecologists, at its recent meeting at Fort Wayne. Dr. Miller is professor of gynecology at Syracuse University, and is a teacher and operator of renown. The association is fortunate in its choice.

---

Dr. H. H. Glosser, of Buffalo, leaves this September for a three-month course of study in the Vienna hospitals.

Dr. H. D. Walker, of Buffalo, announces the removal of his offices from 735 Main street to 915 Main street.

Dr. Herbert D. Pease, of New York, announces his resignation as Director of the State Hygienic Laboratory, New York State Department of Health, and his association with the Officers of the Lederle Laboratories, Consulting Experts in Applied Chemistry, Bacteriology and Sanitary Science, as Director of the Department of Bacteriology.

Dr. F. W. Burkhardt, of Buffalo, soon is to become the physical director of the Bradford, Pa., Y. M. C. A., and will enter upon his duties there within a few days.

Dr. Burkhardt has had charge of the playground work in Buffalo, during the past summer.

Some years ago Dr. Burkhardt was identified with the Y. M. C. A. work in Wilmington, N. C., and besides being a well-known physical director, he has been licensed to practise medicine in New York, New Jersey, Massachusetts and Ohio.

Dr. and Mrs. Herbert U. Williams who have been spending their honeymoon in Switzerland, have returned to Buffalo, and are at No. 221 North street.

Dr. Lewis S. McMurtry, of Louisville, delivered the address on the part of the Americans at the ceremonies incident to the visit to the Washington Monument at Budapest, August 31, 1909. The official program of the Sixteenth International Medical Congress, announced that the American delegation would visit the monument, and it became one of the most delightful incidents of the visit of American physicians to Budapest.

Dr. Ralph Waldo Lobenstine, of New York, by invitation of the president, Dr. William H. Humiston, delivered an address before the American Association of Obstetricians and Gynecologists, at Fort Wayne, September 21, 1909, his subject being "Ruptures of the Uterus during Labor" and was based on seventy-eight cases.

